# Homœopathy and Allopathy? Or Large, Small, and Atomic Doses

**Published:** 1836-10-01

**Authors:** 


Homœopathy and Allopathy? or, Large, Small, and Atomic Doses? By *David Uwins*, M.D. Octavo, pp. 34. Renshaw, Strand. August, 1836.

So our honest friend David has become a convert to Homœopathy! The first thing that struck us in this remarkable pamphlet, was the note of interrogation—Homœopathy *and* Allopathy? If *and* be changed into *or*, the title is intelligible—as it stands, it is rather ambiguous. The worthy Doctor informs us that, some "five and twenty years since," he occupied some of his leisure hours, and "added a little to his slender coffers" by the "sickening trade of book criticising." If we are not mistaken, Dr. U. has been at this trade much within twenty-five years, as the pages of the "Medical Repository could testify. Be that as it may, Dr. Uwins then wrote *against* phrenology, and is now a warm advocate of the doctrine. We would ask Dr. U. if he had studied phrenology when he wrote against it?

Homœopathy is thus very logically and classically defined by our author. "Like loves like, or birds of a feather do well together." Sincerely do we hope that the homœopathic neophyte will "feather *his* nest," and "add a little to his slender coffers," by deserting the allopathic, and joining the "atomic" flock!

"The design of this tract is merely limited to an announcement (upon the strength of *a few* recitals) of the high value the *similia in similibus* theory of medicine has proved in *imparting a power to the medical practitioner which he never possessed before*, and in helping to take off that weight from the mind of a conscientious physician which must necessarily connect itself with public appreciation beyond conscious desert." 7.

So then "*a few recitals*" are sufficient, in the mind of a grave physician, to establish a new theory founded on the ruins of all preceding doctrines and practices!! The archness of sly-boots will be seen in the sequel.

Dr. Uwins refers those of his readers who are desirous, "at very little expense and trouble," to make themselves acquainted with homœopathy, "to consult a *lively* and ingenious tract by the Rev. Thomas Everest"!! This deadly lively tract, and the work of the Rev. Dr. Townsend, he considers as little less than the Alpha and Omega of medical science! Cunning rogue!

"I *prose* in this manner for a particular, *a specific, and a selfish purpose;* and while in the execution of the duty I impose upon myself on the present, as on all occasions, to say what I think, I may say some things which will not sound pleasant to allopathic 'ears polite.'" 9.

Surely Dr. Uwins will be the death of us. Fortunately the cessation of cholera prevents the worthy Doctor from being the death of some who would not be able to laugh at the business.

> "I was about being cruel enough to confess that I almost wish to see a few cases of cholera re-appear, in order to have an opportunity of proving the efficacy of the practice now referred to, principally, in the first instance, being constituted of very small and frequently-repeated doses of camphor." 11.

It appears that Dr. Uwins lent a deaf ear to the new doctrine until he accidentally heard "from very many individuals," that homœopathy was gaining ground with the *public*—and that—"*alas! allopathic carriages would soon be at a low discount.*" This intelligence probably reminded him of the heavy bill of his coachmaker in Long Acre, and, like a knowing one, he considered it prudent to sell out his shares in ALLOPATHY, and embark his capital in the ATOMIC speculation.

> "Surely, under all these circumstances—which I have thus briefly and *most candidly* narrated—I cannot be accused of being 'blown about by every wind of doctrine' that passes by, in having come to the determination of modifying my own practice by an infusion of the new principles." 12.

Oh, not at all, dear Doctor. You have not been "blown about"—you have merely "blown the gaff," as the sailors would say, on your homœopathic friends.

> "I have come to the conclusion that homœopathy, be it true or *false,* deserves the thanks of all thinking and thankful men, for the information it has given us respecting the power possessed by doses of medicine *(prepared in a particular way)* and divided to an extent almost unbounded." 14.

Aye, aye, "*prepared in a particular way*"—known only, of course, to the initiated! Most *naively* does the Doctor add—

> "This is a *principle* which pervades the whole *scheme* of homœopathic agency."

Not a doubt of it. The homœopathist either prepares and dispenses his own atomic doses, in his own way—or insists that they be prepared by a particular homœopathic pharmacopolist—and thus the "scheme" works well—for the discount of "allopathic carriages."

Dr. Uwins began the practice of homœopathy on himself—and we have no doubt that the experimenter had full confidence in—the *innocence* of the remedies.

> "A two-grain dose of James' powder was lying in my bed-room for intended use; and it occurred to me to take up by a moistened finger an unappreciable quantity, which I put on my tongue, and in the night I broke out into a general and profuse perspiration." 15.

We were going to quote Scripture in proof that FAITH will work wonders, when we remembered that it required the size of a "grain of mustard seed" to effect a miracle—and consequently that the dose in this case was *allopathic,* and not applicable to the subject. That the Doctor did sweat, we are bound to believe; but, as he does not inform us whether it was in the month of August or January that his pores broke loose, we, allopathic sceptics, have some hesitation in attributing the "profuse perspiration," to an "unappreciable quantity" of the pulvis Jacobi.

But we must proceed to experiments on others as well as on self.

A lady living near Woolwich "had been beset *by* years with a full chorus of anomalous maladies—acid secretions from the stomach, for which she had taken soda enough almost to neutralize all the acid contained in Apothecaries' Hall—pains and low spirits, sallow complexion, &c. &c." "In a word, diet and medicine had been *aimed and aimed*, again and again, at her poor sensitive body and mind, so that the task of renovating seemed almost hopeless." But Dr. U. did not then know the miraculous powers of "atomic doses." He gave the lady the *forty-eighth part of a grain* of blue-pill, "which roused and excited the *functional dynamics* to such an extent as to prove more sensible in its agency than anything she had ever before taken" !!! This fellow will be the death of us!

Dr. U. avers that imagination had nothing to do with this supernatural agency. "She expressed herself as being sensible that the right nail was now hit, and that I had only to continue my blows to be more successful than I had hitherto been with her."

Some of our readers may shrug up their shoulders and cry "*credat Judæus*." But there is a very easy and natural solution of the enigma. Everything the Doctor had given this lady, previously, made her worse. He gave her the atomic dose of blue-pill—that is to say, he gave her nothing; and the poor lady felt the benefits of homœopathy.

*Case* 2. A lady had been teazed for months with bronchial cough which set time and medicine at defiance. She was cured by a single dose of medicine, consisting of the twelfth part of a grain of extract of aconite, and the same of ipecacuanha. The Doctor seems to have shuddered at the prodigious dose he gave his patient. "The twelfth part of a grain is of *enormous* amount," for this lady was troubled afterwards with headaches, which were, no doubt, owing to this departure from the strict rules of homœopathy."

*Case* 3. Dr. U. was called to a gentleman in Bond Street by "one of the most intelligent men in our profession." The patient laboured under "a sort of nervous fever, connected with, or dependent on, cerebral irritation." The Doctor applied *leeches* which, according to homœopathic canons, "are worse than vain things"—and ordered the specific—the 12th part of a grain of aconite. He also ventured on a quarter of a grain of blue-pill. "His case very shortly assumed a most pleasing aspect," and the patient told the apothecary—"This doctor of your's is certainly a most *penetrating* and able man—a position to which I was very ready to subscribe."

*Case* 4. A young lady laboured under suicidal mania. She took a homœopathic dose of *aurum*, which was repeated after the lapse of a week. She is now "in a condition promising thorough recovery."

We will quote only one more case.

*Case* 5. "Miss ———, Pentonville, whose sister had died of consumption,

To this young lady I ordered medicines so exceedingly minute, according to admitted principles, that fearing her medical attendant would think I had committed a mistake, I wrote at the corner of the prescription "Homœopathy," which my friend Quin would not have allowed me to be correct in doing. This young lady paid her third visit to me yesterday morning, with the pallidness of her cheek exchanged for the bloom of health." 24.

We read over this case twice, in search of the disease for which the Doctor prescribed these exceedingly minute doses of medicine—but in vain. All we could make out was—that the patient's *sister* died of consumption !!

By this time our readers are beginning to exclaim that HOMŒOPATHIA is HOMŒO-HUMBUG—and that Dr. Uwins is a homœo-maniac. The first is true—the second is erroneous. Dr. Uwins is not mad. The whole pamphlet is a most "ingenious device"—a homœopathic "encyclical"—portraying the principles and practice of the new doctrine so ingeniously that many people would think him *in earnest*, though misled and deceived. But it is no such thing. From the days of Gulliver to M'Gee—from the witty traveller of Lilliput to the witless bigot of Exeter Hall, there has not been put forth a more clever hoax than this brochure of Dr. Uwins. It exhibits a most faithful though fictitious picture of homœopathy, under the mask of reality. Up to the very last moment, Dr. Uwins maintains the *assumed* character of homœopathist, with admirable skill. *Voila.*

"Although here I do not intend to theorize, I will just intimate that an electric principle is supposed to mix with the medicinal ingredient—to be infused into the ingredient by the mode of its admixture with the vehicle—and greatly to aid the operative virtue of the atoms." 31.

In fine, though long acquainted with the author of this pamphlet, and entertaining a high opinion of his talent for humour and sly satire, yet we had no idea of the powers which he has here exhibited of exquisite irony. Dr. Uwins has, indeed, "hit the right nail on the head"—and performed the office of undertaker to Homœopathy, with as much dexterity as mercury does to the flitting shades of mortals.

"Tu pias lætis animas reponis
Sedibus, virgâque levem coerces
Aurea turbam—superis deorum
Gratus et imis."—*Hor.*

